# Effect of *N′*-nitrosodimethylamine on red blood cell rheology and proteomic profiles of brain in male albino rats

**DOI:** 10.2478/v10102-011-0020-z

**Published:** 2011-09

**Authors:** Areeba Ahmad, Ravish Fatima, Veena Maheshwari, Riaz Ahmad

**Affiliations:** 1 Biochemical and Clinical Genetics Research Laboratory, Section of Genetics, Department of Zoology, Aligarh Muslim University, Aligarh, India; 2 Department of Pathology, Jawaharlal Nehru Medical College, Aligarh Muslim University, Aligarh, India

**Keywords:** hepatic fibrosis, hypoxia, N′-nitrosodimethylamine (NDMA), proteomic profiles, RBC rheology

## Abstract

We investigated the effects of *N'*-nitrosodimethylamine (NDMA) induced toxicity on red blood cell rheology in male rats and identified bands in proteomic profiles of brain which can be used as novel markers. Polyacrylamide gel electrophoresis (PAGE) profiles exhibited constitutive as well as induced expression of the polypeptides. Remarkably, the molecular weight range of the polypeptides (8–150 kDa) corresponded to that of the family of heat shock proteins. Our results revealed significant changes in blood parameters and showed the presence of acanthocytes, tear drop cells, spicules and cobot rings in the treated categories. Lactate dehydrogenase and esterase zymograms displayed a shift to anaerobic metabolism generating hypoxia-like conditions. This study strongly suggests that NDMA treatment causes acute toxicity leading to cell membrane destruction and alters protein profiles in rats. It is therefore recommended that caution should be exercised in using NDMA to avoid risks, and if at all necessary strategies should be designed to combat such conditions.

## Introduction

*N′*-nitrosodimethylamine (NDMA) is a potent hepatotoxin and carcinogen. Occupational exposure to NDMA may happen in a large number of places including industries such as tanneries, pesticide manufacturing plants, rubber and tire manufacturing plants, alkylamine manufacture/use industries, fish processing industries, foundries, and dye manufacturing plants (Mitch *et al.*, 2003). Under certain conditions, NDMA may be found in outdoor air, surface waters (rivers and lakes, for example), and soil (ATSDR, 1989; Mitch *et al.*, 2003). Most reactions require a source of nitrite and a secondary, tertiary or quaternary amine to form NDMA (Smith & Loeppky, 1967; Fiddler *et al.*, 1972; Kimoto *et al.*, 1981).

It is widely accepted that NDMA induces hepatic fibrosis and cirrhosis (Haggerty & Holsapple, 1990), and the toxicity is caused by intermediary metabolites produced during metabolism of NDMA rather than by the parent compound itself. The relatively safe concentrations vary in different animals, *e.g.* in rats the oral LD_50_ of NDMA has been reported to be 40 mg/kg body weight (ATSDR, 1989). Other compounds which similarly to NDMA induce hepatic fibrosis in laboratory animals are: nitrosodiethylamine (NDEA), thioacetamide and carbon tetrachloride (George & Chandrakasan, 2000; Shimizu *et al.*, 2001; Palacios *et al.*, 2008; Smyth *et al.*, 2009; Karantonis *et al.*, 2010). The available literature suggests that NDMA-induced hepatic fibrosis in rats is a very successful and reproducible model for various clinical and toxicological studies. Hepatic fibrosis (HF) is a pathological condition in which abnormal, tough and non-functional fibrous connective tissue, especially mature collagen fibers, accumulates in the extracellular matrix (ECM) (Wells, 2005). The imbalance between ECM synthesis and its degradation causes stimulation of inflammatory immune cells to secrete cytokines, growth factors and other molecules to activate hepatic stellate cells (HSCs). The activated HSCs, being proliferative and fibrogenic, express α-smooth muscle actin and various other connective tissue proteins such as collagen types I, III and IV to cause hepatic fibrosis (Friedman, 1993; Pinzani & Marra, 2001; Lotersztajin *et al.*, 2005). Induction of fibrosis by NDMA causes hypoalbuminemia, due to increase in urinary excretion of protein catabolites (George & Chandrakasan, 2000), and it alters activities of various enzymes such as LDH, ALP, AST, ALT (Ahmad *et al.*, 2009a).

It has also been reported that increased oxidative stress is involved in the pathogenesis of hepatic fibrosis (Jenkins, 1985). The higher generation of free radicals by NDMA is a contributor to the increased oxidative stress (Jenkins, 1985) that causes lipid peroxidation, which may decrease fluidity of lipid phase of biomembranes. Since red blood cell and nerve cell membranes are rich in polyunsaturated fatty acids, both of these cell types are assumed to be highly sensitive to the peroxidative process caused by NDMA.

We previously demonstrated that NDMA induced hepatic fibrosis in rats within 21 days, which could be reversed following administration of *Operculina turpethum* extract (Ahmad *et al.*, 2009a). The present attempt aimed at identifying changes in proteomic profiles of soluble proteins of rat brains produced due to toxicity generated by NDMA. We identified proteins and polypeptides which could serve as novel and convenient markers of monitoring *in vivo* toxicity. No previous study has dealt with *in vivo* toxic effects of NDMA on brain polypeptides and RBC rheology in the rat. Effects on RBC membrane and induction of hepatic fibrosis have been taken as known markers of toxicity evaluation for a variety of chemical compounds, including NDMA (Brkic *et al.*, 2008; Fetoui *et al.*, 2008; Ahmad *et al.*, 2009a). Additional markers which substantiate these findings are: lactate dehydrogenase (LDH) and esterases (Est). These are important respective enzymes required for proper glucose metabolism in the brain and for motor functioning.

## Materials and methods

### Chemicals and reagents

Acrylamide, *N*′-nitrosodimethylamine (NDMA), nicotinamide adenine dinucleotide (β-NAD), nitro blue tetrazolium (NBT), phenazine methosulphate (PMS) were purchased from Sigma-Aldrich. α- and β-naphthylacetate, giemsa stain were purchased from Qualigens Fine Chemicals, India. All other chemicals and reagents used were of analytical grade.

### Animals

Adult healthy male albino rats of the Wistar strain, 7–8 weeks old weighing around 145±10 g, were used for experiments. The rats were housed in well aerated polycarbonate cages with proper humane care at the animal house facility in the department, with light: dark exposure of 12:12 hrs. The rats were acclimatized for a week and fed regularly with sterilized diet and water available *ad libitum*.

### Induction of hepatic fibrosis

The animals were divided into two groups comprising fifteen rats each. One group received intraperitoneal injections of NDMA in doses of 1 mg/100 g body weight (10 µL diluted to 1 mL with 0.15 M sterile NaCl), while the other group served as control and received the same amount of 0.15 M sterile NaCl through intraperitoneal injections. The injections were given on the first three consecutive days of each week for a period of three weeks without anesthesia. Three rats from each group were sacrificed on days 4, 7, 11, 14 and 21 from the start of the experiment. The experimental animals were procured and sacrificed according to the University Ethical Regulations.

### Blood smear preparation

Freshly collected blood was taken and divided into two halves. One half was used for blood cell counts and determination of hemoglobin, while the other half was used for preparing permanent smears of RBCs for further investigation. The slides were fixed in methanol for 12–15 min and stained with Giemsa for 8–10 min. These slides were fixed in DPX and randomly selected for rheological studies.

### Assessment of hepatic fibrosis

Hematoxylin and eosin (H&E) staining of liver sections (5 µm) was done to assess the development of hepatic fibrosis histochemically. Stained slides were examined and photographed under Nikon microscope with an LCD attachment (Model: 80*i*).

### Brain homogenate preparation

The brains were immediately dissected out in sterilized condition avoiding contamination of neighboring tissues. They were weighed and homogenized in pre-chilled 50 mM Tris-HCl buffer (pH 7.5) maintaining tissue to buffer ratio 1:4 (w/v). The homogenates were centrifuged at 12,000 rpm for 15 min. The clear supernatants were processed for protein determination and stored in different aliquots with equal volumes of glycerol containing 5 mM PMSF at −20 °C for further analysis.

### Protein determination

Protein concentration in different brain homogenate samples of controls and treated animals was determined by the method of Bradford (1976) using Coomassie Blue as color reagent and bovine serum albumin as standard. Optical density (absorbance) was taken at 595 nm on a UV-1700 Pharma-spec UV-Visible spectrophotometer.

### Protein profiling of brain homogenates

For non-denaturing polyacrylamide gel electrophoresis (PAGE), the protocol of Laemmeli (1970) was followed with the modification that the gels were lacking SDS. Gels were 7.5% in acrylamide containing 15% glycerol. Equal amounts of protein were loaded in each well on polyacrylamide gels and the runs were made for 5 hrs at 9 V, 35 mA/gel. Staining of gels was done in Coommassie Brilliant Blue for the identification of protein bands followed by destaining in 5% glacial acetic acid.

### Molecular weight determination of polypeptides and their localization by silver staining

The process was done in denaturing condition where the PA gels, running buffers and samples had SDS in their known molarities (Laemmli, 1970). The gels were run for three hrs at 16V, 60mA/gel at room temperature. After the run was over, the gels were directly processed for silver staining (Nesterenko *et al.*, 1994). Replica gels were also run under similar conditions and used for CBB staining after overnight wash in 5% acetic acid.

### Visualization of lactate dehydrogenase (LDH) and esterase (Est) isoenzymes

Electrophoresis was carried out according to the protocol described previously (Ahmad & Hasnain, 2005). For LDH staining, gels were incubated in reaction mixture containing substrates, intermediates and coenzymes in their evaluated concentrations (Ahmad *et al.*, 2009b). Gels for Est staining were separately incubated in reaction mixture containing each α-naphthyl acetate and β-naphthyl acetate (5.58 × 10^−3^ mM) as substrates at 25 °C.

### Documentation, densitometry and quantitative assessment of PAGE profiles

H&E stained sections of liver biopsies were visualized and photographed under microscope (Nikon, Model: 80i) with in-built microprocessor. Blood smear slides were photographed under microscope (Nikon, Model: 80i) attached to camera (Nikon).

Stained PA-gels were documented using SONY-CYBERSHOT digital camera (Zoom-4×, 12 Megapixels) and by direct scanning on an all-in-one HP Deskjet (F370) computer assembly. Data from both records were used for further processing and analysis. Gel-scans were processed through Adobe Photoshop (version 7.0) to obtain the best contrast for densitometric analysis through software. Densitometry of the selected gel-scans was done using Scion Imaging (Scion Corporation; Beta release, 4.0) and GelPro (Media Cybernetics, USA) software programs.

### Statistical analysis

The values of all blood-related parameters of control and treated specimens were compared and presented as mean±SD (*n*=5). To test the significant differences among the obtained values and enzyme activities, Student's *t*-test was applied at *p*<0.05.

## Results

**Figure 1:**
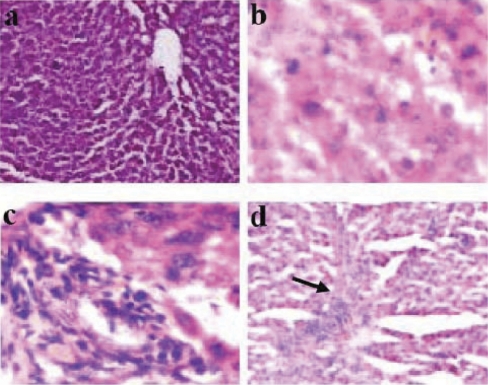
Hematoxylin and eosin (H&E) staining of rat liver sections during the pathogenesis of NDMA-induced hepatic fibrosis (a) Control liver (×125). (b) NDMA, day-7 (×250). Severe congestion and hemorrhagic necrosis. (c) NDMA, day 14 (×250). Severe neutrophilic infiltration and fatty changes. (d) NDMA, day 21 (×125). Marked hepatic fibrosis (arrow) and deposition of collagen fibers.

Toxicity due to NDMA treatment was assessed by the *in vivo* induction of hepatic fibrosis for 21 days. It was confirmed by H & E staining of liver biopsies. Stained liver sections of rats showed disruption in normal liver architecture, inflammation, hemorrhage and fibrosis with distinct deposition of thick collagen fibers within 21 days of NDMA treatment (Figure 1).

**Figure 2:**
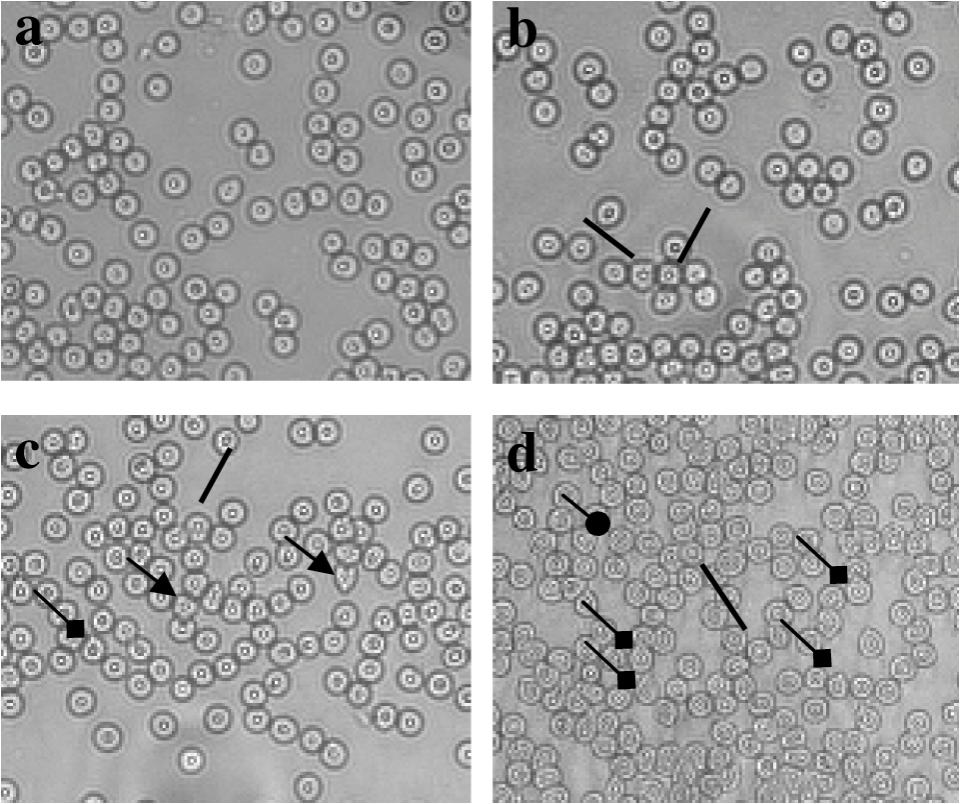
Plates showing red blood cells of rats stained with Giemsa. (a) Normal morphology of RBC in control samples (×600). NDMA-treated samples demonstrated visible membrane destructions and presence of different cell shapes. (b) Spicules, crenated (—). (c) Spicules and acanthocytes (→). (d) Spicules, dacryocytes (tear drop, —♦) and cobot rings (—•).

Table 1 demonstrates changes in peripheral blood of rats treated with NDMA for 21 days, details are given under Materials and Methods. Significant increase in hemoglobin, total leukocyte count (TLC), neutrophils and hemolyzed RBC count (*p*<0.05) can be observed. Similarly, a significant decline in the levels of lymphocytes of the order of 17% as compared to controls and intact RBC count is recorded (*p*<0.05). Stained RBC smears show the presence of acanthocytes, dacryocytes (tear drop cells), spicules (crenated) and cobot rings in treated samples indicating anemia and liver dysfunction (Figure 2). Increase in the number of these cell types was time and dose dependent.

**Table 1:** Some blood-related parameters in control and NDMA-treated samples of rats.

Parameter	Control	Treated
Hemoglobin (gm %)	5.7±0.95	8.0±1.02*
Total leukocyte count (m/cu mm)	3170±109	37190±145*
Differential leukocyte count		
Neutrophils (% )	19±1.8	38±2.07*
Lymphocytes (%)	74±3.69	57±3.88**
Eosinophils (%)	02±0.11	0
Monocytes (%)	04±0.31	05±0.44
Basophils (%)	01±0.2	0
Intact RBCs count (10^6^ cells/mL)	7.2±1.11	5.9±0.98**
Hemolyzed RBC (%)	2.5±0.19	48±3.04*

The obtained values are expressed as mean±SD (n=5).

* *p<*0.05, in control and treated samples.

** *p<*0.01, in control and treated samples.

**Figure 3:**
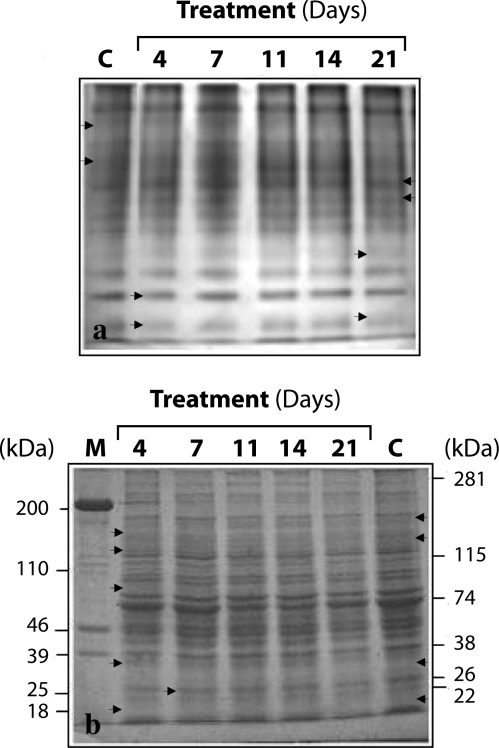
Typical polyacrylamide gel electrophoretic (PAGE) profiles of brain homogenates (a) native PAGE: C, Control samples (untreated); 4, 7, 11, 14 and 21 represents days of NDMA treatments given to the rats. (b) Protein profiles under denatured conditions (SDS-PAGE): M, Molecular weight marker (chicken actomyosin); C, control samples; 4, 7, 11, 14 and 21 represent days on which NDMA was administered to rats. Arrows (→) indicate the peptide (s) showing differences.

Native-PAGE profiles showed the presence of nineteen protein bands in soluble brain proteins of the control and treated samples (Figure 3a). Densitometric analysis of gel-scans revealed quantitative differences in protein band # 3, 6, 7, 9, 15, 17, 18 and 19 (shown with arrow heads). Their expression was time-specific and correlated with changes in the stages of pathogenesis. Representative profiles in SDS-PAGE revealed the existence of thirty-seven and thirty-nine polypeptides in the respective control and treated samples of soluble brain proteins (Figure 3b). Three polypeptides of the molecular weights 184, 139, and 31 kDa observed in control samples were totally absent at any stage of treatment with NDMA. In SDS-PAGE profiles of brain, six novel polypeptides of the molecular weights 147, 123, 81, 33, 25 and 18 kDa were detected in the treated groups (Figure 3b). A novel stage-specific polypeptide (M*r*=25 kDa) was detected in a significant amount (*p*<0.05) on day 4 of the treatment and at following stages.

**Figure 4:**
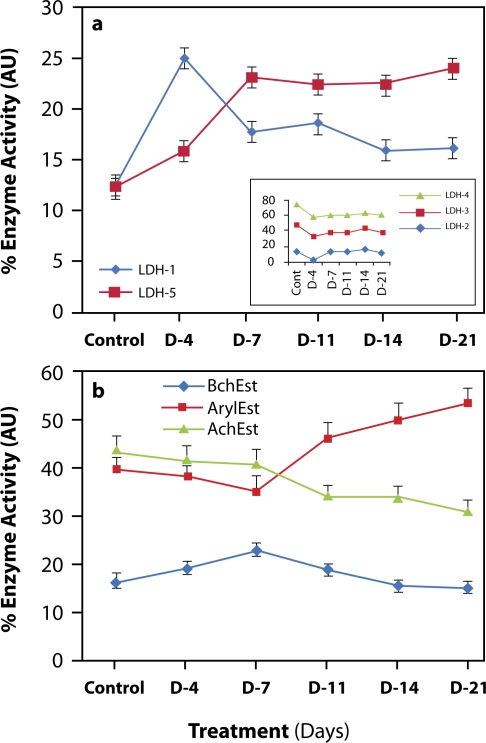
Graphical representation of the changes in enzyme activities in brain of rats during progression of hepatic fibrosis. (a) LDH and, (b) Esterases.

During the present study, we compared zymograms of lactate dehydrogenase (LDH) and esterases (Est) in the soluble fraction of brain samples of control and treated animals. In the control samples, the isoenzyme rank was LDH-3>LDH-4>LDH-1>LDH-5>LDH-2. On day 4 of NDMA treatment, significant changes in isoenzyme levels were noted that did not alter the ranking and order of preference of LDH isoenzymes in the tissue investigated. On day 7 of treatment, abrupt up-regulation in the activity of LDH-5, concomitant with down-regulation of LDH-1 and -4, was noted in treated fractions (Figure 4a). This switchover persisted up to the end of treatment and led to a change in the overall rank of LDH isoenzymes: LDH-3>LDH-5>LDH-4>LDH-1>LDH-2. No apparent change in LDH heterotetramers of brain was detected during the treatment and induction of fibrosis (Figure 4a, inset).

Typical zymograms of esterases of control and NDMA-treated group of rats demonstrate the presence of butyryl cholinesterase (BchEst), paraoxonase/arylesterase (ArylEst) and acetylcholinesterase (AchEst) in the brain. Relative quantities of AchEst to ArylEst show a significant decline (*p*<0.05) (Table 2) and display a typical shift in the levels of AchEst to ArylEst after day 7 in the treated group of rats (Figure 4b).

**Table 2:** Values showing the relative presence of acetylcholine esterases and aryl esterases in the brain of control and NDMA-treated rats.

Esterases	Control	Day-4	Day-7	Day-11	Day-14	Day-21
ArylEst	39.7±2.88	38.66±2.2	35.77±2.92	46.38±3.41	50.12±3.92	53.54±3.56
AchEst	43.9±3.01	42.08±2.99	41.26±2.87	34.64±2.2	33.92±2.6	31.15±2.43
AchEst: ArylEst	1.1057	1.0884	1.1534	0.7468	0.67677*	0.581*

The data are represented as mean ± standard deviation (n=5).

* *p<*0.05, in control and treated samples.

## Discussion

We had previously shown that *N'*-nitrosodimethyl-amine (NDMA) treatment in rats in the specified concentrations induced hepatic fibrosis (HF) within 21 days (Ahmad *et al.*, 2009a). During the present investigations, HF was again successfully induced by NDMA in rats treated for 21 days under identical conditions. Hematoxylin and eosin staining of liver sections confirmed changes characteristic of intensive HF, *i.e.* inflammation, disruption of normal liver architecture, hemorrhage and distinct deposition of thick collagen fibers (Figure 1). In a search for easy monitoring of biochemical markers for early neurotoxicity detection, apart from RBC related parameters and the enzyme levels, we focused on electrophoretic profiles of proteins/polypeptides with increasing duration and course of HF to help identify participant(s) in the molecular cascade of the neurotoxic stimulus of the insult (Haggerty, 1990; Jellinger, 2001).

Our findings in this investigation established a correlation between NDMA-induced HF and changes in clinical blood parameters and brain polypeptides. In NDMA-treated rats, the intact RBC counts decreased while hemolyzed RBC count, hemoglobin, TLC, and neutrophils increased significantly (*p*<0.05). Thus, the blood parameters strongly indicated acute toxicity in NDMA-treated rats. Oxidation of NDMA within RBCs may have led to free radical generation and hemolysis, which consequently caused hemoglobin release exerting a multitude of toxic effects (Everse & Hsia, 1997; Armutcu *et al.*, 2005). One of the most important effects highlighted by these authors is the liberation of iron from RBCs. Iron ion plays an important role as a redox catalyst and its liberation will therefore increase the total pro-oxidant potential. Lipid peroxidation mediated by free radical generation is believed to be an important cause of damage to cell membranes, since polyunsaturated fatty acids of the cellular membranes are degraded by free radicals that ultimately disrupt membrane integrity (Armutcu *et al.*, 2005).

In native PA gel profiles, the total number of protein bands in soluble brain proteins of control as well as the treated groups was nineteen (Figure 3a). However, few polypeptides showed significant quantitative differences (*p*<0.05), depending also on the duration of NDMA treatment. Four categories were recognized: (i) gradual quantitative decrease in band #3 and 6 corresponding with the increasing duration of NDMA treatment; (ii) abrupt increase in bands #7 and 9 on day 21 of treatment; (iii) sudden increase in bands #15 and 18 on day 4 followed by a gradual decrease up to day 21 of treatment; and, (iv) rapid decrease in band #17 and 19 on day 4 followed by a continuous increase up to day 21 of treatment. It is thus obvious that HF induction by NDMA brings about quantitative changes in soluble proteins of the treated rat brains, which indicates their diagnostic value. Because of the concomitance with the changes in RBC rheology, which are an accepted marker of toxicity, the quantitative changes observed in brain protein bands are also indicators of toxic effects of NDMA.

Quantitative changes in protein profiles can occur not only at transcriptional and translational levels but disturbances in folding and assembly of polypeptides may also appear. We therefore analyzed NDMA-treated samples by SDS-PAGE and detected a total of 37 polypeptides in the control group and 39 in the treated samples, However, the differences were more pronounced in terms of molecular weights: (i), four polypeptides with M*r* 184, 139, 31 and 17 kDa were specific to control samples (ii), six polypeptides of M*r* 147, 122, 81, 33, 25 and 18 kDa were specific to treatment groups (iii), a single polypeptide of M*r* 25 kDa showed temporal expression from day 4 of treatment onwards (Figure 3b). Cells display stress-response characterized by induction of a variety of proteins, including proteins of the heat shock family in response to environmental and pathophysiological stimuli (Hightower & White, 1981; Brown, 1994; Richter-Landsberg & Goldbaum, 2003). Many of the gene products are induced by a variety of injurious stimuli belonging to the stress-protein family and fall within the molecular weight range of 8–150 kDa (Gonzalez *et al.*, 1991; Benjamin and McMillan, 1998; Richter-Landsberg and Goldbaum, 2003). We suggest that NDMA-induced injury in rats limits oxygen carried to brain cells because of a decrease in intact RBC count. This would produce a stressful (hypoxic) condition resulting in induction and expression of proteins with M*r* 93, 47and 33 kDa, which belong to the heat shock protein (*hsp*) family. However, as reported by previous workers, constitutive protein forms of M*r*=100, 72 and 27 kDa were also present (Hightower & White, 1981; Brown, 1994; Richter-Landsberg & Goldbaum, 2003). Although the function of all the proteins belonging to the *hsp* family is not yet known, it is assumed that constitutive forms or molecular chaperones are important in post-translational processing, such as protein folding, transport between organelles and proteolysis (Mayer & Brown, 1994; Massa *et al.*, 1996; Benjamin & McMillan, 1998). It is also likely that the three inducible forms detected in electrophoretic profiles of soluble brain proteins in NDMA-treated rats have similar functions and participate in stabilizing and refolding partially denatured proteins or promoting their degradation (Nagata & Hosokawa, 1996; Pratt, 1998; Landry & Huot, 1999).

Some reports have shown that nitroso compounds create a hypoxic environment (Jenkins *et al.*, 1985; Haggerty & Holsapple, 1990). The significance of lactate dehydrogenase isoenzymes as indices of hypoxia/anoxia in various vertebrates has often been emphasized (Singh & Kanungo, 1968; Jakob *et al.*, 1993; Crawford & Davies, 1994; Ahmad & Hasnain, 2005). In NDMA-treated rats from day 7 onwards, a switchover of homotetramer LDH-1 (H_4_) to LDH-5 (M_4_) was noticed in the treated animals (Figure4a). Similarly, a shift of acetylcholinesterases (AchEst) to arylesterases (ArylEst) was also observed in brain extracts of NDMA-administered rats (Figure4b). Since an increase in LDH-5 (M_4_) indicates hypoxic stress, it is obvious that NDMA generated a hypoxia-like condition in brain cells. Moreover, the coexistence of LDH-1 with AchEst indicates that LDH-1 is present in nerve fragments due to entrapped cytoplasm in the axon and is probably consumed by these cells (Johnson, 1960; Benzi, *et al.*, 1980; Laughton, *et al.*, 2000).

In light of the above findings it can be concluded that RBC hemolysis, abnormal protein profiles and a high degree of enzymatic variations in the brain are the consequences of NDMA-induced toxicity in rats. In addition, these changes are also associated with the onset of hepatic fibrosis on day 7. Our results showed for the first time that certain protein bands and polypeptides of the soluble fraction of the brain are suitable markers of toxicity produced by NDMA and similar compounds. This finding should help elucidate the detailed mechanism of toxicity by using a combination of molecular biology techniques with proteomic profiles and other parameters, thus extending available experimental models. The outcome will help in designing remedies to treat pertinent conditions.
